# M13 Bacteriophage-Based Sandwich Assay for *Salmonella* Detection: A Dual-Signal Approach Combining Colorimetric and SERS Techniques

**DOI:** 10.3390/foods15203681

**Published:** 2026-10-16

**Authors:** Shengnan Zhan, Yanke Wang, Yihui Yang, Qi Chen, Xizhi Shi, Hui Wang, Wenhua Yang, Xin Zhang, Tao Huang

**Affiliations:** 1 Zhejiang–Malaysia Joint Research Laboratory for Agricultural Product Processing and Nutrition, College of Food Science and Engineering, Ningbo University, Ningbo 315211, China; 2 Jiangxi Provincial Key Laboratory of Bioengineering Drugs, National Engineering Research Center for Bioengineering Drugs and Technologies, Institute of Translational Medicine, Jiangxi Medical College, Nanchang University, Nanchang 330031, China; 3 Key Laboratory of Aquacultural Biotechnology, Ministry of Education, School of Marine Sciences, Ningbo University, Ningbo 315211, China; 4 State Key Laboratory of Food Science and Resources, Nanchang University, Nanchang 330047, China; 5 Key Laboratory of Processing and Nutritional Health of Tropic and Subtropical Aquatic Specialty Foods, Guangzhou 510380, China

**Keywords:** M13 bacteriophage, *Salmonella*, SERS, signal amplification, dual signal

## Abstract

Herein, a dual-modal detection strategy for *Salmonella* using an engineered bifunctional M13 bacteriophage is presented. A *Salmonella*-specific binding peptide was fused to the pIII protein, while a streptavidin (SA)-binding nanotag was displayed on the pVIII protein. Taking advantage of the approximately 2700 copies of pVIII per phage, the addition of SA-HRP enabled massive enzymatic loading, which served as the key to generating both SERS and colorimetric signals in the presence of TMB substrate. Under optimized conditions (37 °C, ~1.5 h), the assay achieved limits of detection of 3.4 CFU/mL for the colorimetric mode and 2.7 CFU/mL for the SERS mode. The method exhibited excellent specificity and demonstrated satisfactory accuracy in complex matrices including *Penaeus vannamei*, beef, and peanuts, highlighting its strong potential for practical application in rapid food safety detection.

## 1. Introduction

*Salmonella typhimurium* is one of the most common foodborne pathogens of global concern, causing illnesses ranging from self-limiting gastroenteritis to severe systemic infections, thereby posing substantial threats to public health and food safety [1]. Conventional detection techniques, including culture-based methods [2], polymerase chain reaction (PCR) [3], and enzyme-linked immunosorbent assays (ELISA) [4], are often constrained by long assay times, labor-intensive procedures, or limited sensitivity, particularly when bacterial concentrations are extremely low. Consequently, there is a compelling demand for rapid, ultrasensitive, and multiplexed detection platforms.

Conventional *Salmonella* detection strategies typically rely on sandwich-type immunoassays using pairs of monoclonal or polyclonal antibodies [5,6]. However, dual-antibody-based approaches are inherently limited by prolonged production cycles, high costs, and extensive screening requirements [7]. Nanobodies have attracted increasing attention as alternative recognition probes owing to their high binding affinity, specificity, structural stability, and amenability to genetic engineering [8]. Nevertheless, recognition capability alone may not guarantee sufficient signal output, potentially limiting detection sensitivity. Therefore, functional materials that integrate specific molecular recognition with intrinsic signal amplification are of considerable significance.

Among various recognition molecules, M13 bacteriophages have attracted considerable attention owing to the dual functionality of the displayed recognition elements, which integrate target-specific binding with signal amplification [9]. M13 bacteriophage is a filamentous virus about 880 nm long and 6.5 nm in diameter [10]. The minor coat protein pIII, present in 3–5 copies, can be genetically engineered to display nanobodies or peptides that specifically recognize *Salmonella typhimurium*. The major coat protein pVIII, comprising approximately 2700 copies, provides abundant N-terminal sites for chemical modification and functionalization [11,12,13]. For example, fusing a gold nanoparticle-binding peptide to pVIII enables dense loading of gold nanoparticles onto a single phage [14]. This dense loading not only enhances plasmon signals but also creates numerous interparticle nanogaps that can act as electromagnetic hot spots for surface-enhanced Raman scattering (SERS) [15]. Thus, an engineered M13 phage carrying gold nanoparticles can simultaneously serve as a recognition element, a colorimetric reporter, and a SERS substrate. This unique feature motivates the development of a dual-mode detection platform.

However, single-modality detection systems may suffer from insufficient reliability in practical applications due to complex matrix interference or instrumental variability [16,17,18]. Dual-mode strategies combining SERS and colorimetric readouts can provide cross-validation: SERS offers high sensitivity (down to the single-molecule level) and fingerprint-like spectral features, whereas colorimetric signals enable rapid visual screening without sophisticated instruments [19,20,21,22]. The mutual cross-validation of these two modes improves detection accuracy and reliability while simultaneously broadening the dynamic detection range to accommodate samples with varying analyte concentrations, thereby enhancing the applicability and anti-interference performance of the method in complex real-world samples.

In this study, a SERS-colorimetric dual-mode sensing platform was established based on a bifunctional M13 bacteriophage for the detection of *Salmonella typhimurium*. A *Salmonella*-specific peptide (VTPPTQHQ) was genetically displayed on the pIII protein, conferring selective recognition capability [23,24]. The pVIII proteins were biotinylated to enable efficient immobilization of streptavidin-conjugated horseradish peroxidase (SA-HRP), which catalyzed the oxidation of TMB to generate colorimetric signals [25,26]. Owing to the high copy number of pVIII (2700 copies per phage), multiple enzyme anchoring sites were provided for signal amplification. Magnetic gold nanocomposites (MBs@CS@AuNPs) were employed as SERS substrates [27], and the oxidized TMB further enhanced the SERS response, enabling simultaneous acquisition of colorimetric and Raman signals (Figure 1). Notably, the use of M13 bacteriophages as a template for constructing SERS substrates not only increases the density of “hot spots” but also provides a biocompatible interface that facilitates bacterial capture. Quantitative analysis of *Salmonella typhimurium* was achieved by monitoring changes in ultraviolet absorbance and characteristic Raman spectral features. This dual-signal detection strategy enables highly sensitive and quantitative determination of *Salmonella typhimurium* in complex samples. Overall, the proposed platform is expected to advance point-of-care diagnostics for foodborne pathogens and to provide a new paradigm for phage-nanoparticle hybrid systems in infectious disease surveillance.

## 2. Materials and Methods

### 2.1. Materials and Reagents

Analytical-grade tetrachloroauric acid was purchased from Sigma-Aldrich (Shanghai) Trading Co., Ltd. (Shanghai, China). Sodium citrate and 1-(3-dimethylaminopropyl)-3-ethylcarbodiimide hydrochloride (EDC) were purchased from Shanghai Titan Technology Co., Ltd. (Shanghai, China). Ethylenediaminetetraacetic acid (EDTA) and polyethylene glycol (average Mn 8000) were purchased from Shanghai Maclin Biochemical Technology Co., Ltd. (Shanghai, China). Horseradish peroxidase (HRP), HRP-labeled streptavidin (SA-HRP) and 3,3′,5,5′-Tetramethylbenzidine (TMB) (two-component color development solution) were purchased from Beijing Solarbio Technology Co., Ltd. (Beijing, China). N-Hydroxysuccinimide sodium salt (NHSS) and Tween-20 were purchased from Shanghai Aladdin Biochemical Technology Co., Ltd. (Shanghai, China). Magnetic beads (MBs) were purchased from Shanghai Allrun Nano Science & Technology Co., Ltd. (Shanghai, China). Skimmed milk powder was purchased from Shenggong Bioengineering (Shanghai) Co., Ltd. (Shanghai, China). Anti-*Salmonella typhimurium* monoclonal antibody (mAb) was purchased from Meridian Biotechnology Co., Ltd (Memphis, TN, USA). Kanamycin sulfate was purchased from Dalian Meilun Biotechnology Co., Ltd. (Dalian, China). PBS buffer (0.01 M, pH = 7.4) was purchased from China National Pharmaceutical Group Chemical Reagents Co., Ltd (Shanghai, China).

The bacterial strains used in this study include a genetically engineered anti-*Salmonella typhimurium* M13 bacteriophage (the pIII protein can recognize *Salmonella typhimurium* and the pVIII protein was modified and fused with a peptide that can recognize SA), *Salmonella typhimurium*, *Escherichia coli* O157:H7 (*E. coli* O157:H7), *Escherichia coli* ER2738 (*E. coli* ER2738), *Pseudomonas*, and *Shewanella*. All strains were sourced from our laboratory.

### 2.2. Instrumentation

The UV-vis absorbance values were measured using a SpectraMax i3 microplate reader (Molecular Devices, Sunnyvale, CA, USA). The morphology of the bacteriophage was observed using a transmission electron microscope (TEM-HT7700, Hitachi High-Technologies, Tokyo, Japan). Raman spectroscopy was performed using a HORIBA JOBIN YVON Raman spectrometer (HORIBA Scientific, Palaiseau, France) equipped with a 785 nm excitation laser.

### 2.3. Preparation of the “Sandwich” Structure of IMBs-Target Bacteria-M13 Bacteriophage

#### 2.3.1. Preparation of the Capture Probe

In total, 50 μg of MBs were added to 150 μL of phosphate buffer (PB, pH 6.0, 0.01 M) and washed using an external magnetic field, discarding the supernatant. This washing step was repeated twice, and the precipitate was then resuspended in 150 μL of PB (pH 6.0, 0.01 M). Subsequently, 2 mg/mL of NHSS and EDC were added, and the mixture was rotated and incubated for 1 h. After the reaction, the MBs were magnetically recovered, followed by two washes with PB (pH 6.0, 0.01 M). The resulting MB precipitate was resuspended in 150 μL of phosphate-buffered saline (PBS, pH 7.4) and subjected to ultrasonication and vortex mixing to ensure complete dissolution and dispersion. Then, 10 μg of capture antibodies were added, and the mixture was rotated and incubated for 2 h. Non-specific binding sites were blocked by adding skim milk, followed by incubation for 4 h. After magnetic recovery, the immunomagnetic nanobeads (IMBs) were washed twice with PB (pH 7.4, 0.01 M) and finally resuspended in 1 mL of resuspension solution.

#### 2.3.2. Enrichment of *Salmonella typhimurium* by IMBs

The prepared IMBs were incubated with 1 mL of 10^5^ CFU/mL *Salmonella typhimurium* with shaking at 220 r/min for 80 min at 37 °C. The IMBs were collected by magnetic separation and washed 3 times with PBS. After resuspension in PBS, the number of captured bacteria (Na, CFU/mL) was determined by the plate counting method. Meanwhile, the total number of *Salmonella typhimurium* cells (N_0_, CFU/mL) was quantified by direct plating onto LA plates without IMB treatment. The capture efficiency (CE) was calculated using the following equation:

(1) CE (%) = Na/N_0_ × 100%

To enhance the capture efficiency of the IMB probe for *Salmonella typhimurium* enrichment, the capture conditions were systematically optimized.

### 2.4. Detection of Salmonella Typhimurium

The magnetic bead-based immunoassay was performed as follows [28]. Briefly, 50 μg of MBs@CS@AuNPs@Ab (CS=Chitosan), M13 phage and *Salmonella typhimurium* solutions with different concentrations were mixed together and incubated at 37 °C for 80 min. After magnetic separation, the unbound sections were discarded; the combined “sandwich” structures were reserved and washed 3 times with PBS. Then, 100 µL of TMB chromogenic solution, which was prepared and used immediately, was added to resuspend the “sandwich” conjugates. After being exposed to 37 °C for 10 min, the color change in the solution was observed (ox-TMB), and the optical density (OD) at 641 nm was recorded. Next, 10 µL of the MBs@CS@AuNPs was added as the SERS substrate and mixed with an equal volume of the ox-TMB solution, then dropped onto the glass slide for SERS detection. The characteristic peak at 1602 cm^−1^ was selected for SERS analysis. All the experiments were conducted in three parallel runs.

### 2.5. Sample Pre-Treatment and Method Validation

Shrimp (*Penaeus vannamei*), beef and peanut samples were acquired from a local market and were confirmed to be bacteriologically sterile by the plate counting method [29]. For analysis, 1 g (or 1 mL) of the sample was homogenized with 9 mL of PBS solution in a sterile centrifuge tube and vortexed for 5 min. The homogenate was centrifuged at 10,000 rpm and 4 °C for 10 min. After discarding the precipitate and the upper fat layer, the supernatant was transferred to a new tube. Subsequently, 3 mL of the processed sample was spiked with a single colony of *Salmonella typhimurium* and inoculated into 27 mL of fresh LB liquid culture medium. The mixture was incubated at 37 °C with shaking at 250 rpm for 14 h. Aliquots of 1 mL were collected every 2 h for analysis by ELISA-SERS and plate counting.

### 2.6. SERS Detection

The mechanism for the generation of the SERS signal by MBs@CS@AuNPs nanoparticles is shown in Figure 1. Equal volumes (10 µL each) of the MBs@CS@AuNPs nanomaterials and TMB solution were mixed and incubated together for 30 min. Then, the nanomaterials were aggregated under an external magnetic field and then dried. Raman spectra were acquired using a 785 nm laser excitation source with a power of 10 mW and collected through a 50× objective lens with a relatively long working distance. Each SERS spectrum was recorded with an integration time of 30 s. The measurement was carried out by randomly selecting three points on a silicon wafer and scanning over the spectral range of 800–1800 cm^−1^. The characteristic Raman peak was observed at an average wavenumber of 1602 cm^−1^. For actual sample analysis, 10 μL of the sample was processed following the same procedure as described above.

## 3. Results and Discussion

### 3.1. Detection Principle of IMBs-Bacteria-Phage “Sandwich” Structure-Based Colorimetric–SERS Double Signal

This study presents a dual-signal detection platform based on the M13 bacteriophage, which integrates SERS and an ELISA colorimetric assay for the specific detection of *Salmonella typhimurium*.

Magnetic separation and detection: Anti-*Salmonella typhimurium* antibodies were immobilized on MBs, enabling efficient capture and magnetic separation of the target bacteria from complex samples using an external magnetic field. This step effectively reduces background interference and improves detection accuracy [30,31]. Notably, the phage-bacteria complex formed in the first step further enhances signal transduction efficiency.

M13 bacteriophage modification and binding: The M13 bacteriophage was genetically engineered to display a targeting peptide (VTPPTQHQGGGS) specific to *Salmonella typhimurium* on the pIII protein [23,24]. Upon binding to *Salmonella typhimurium*, the engineered M13 bacteriophage forms a stable “sandwich” complex with the target bacteria.

Signal amplification and detection (Figure 1): an SA-binding peptide (MDVEAWLGAR) was fused to and expressed on the pVIII protein, subsequently conjugating with SA via SA-HRP to generate an enzyme-labeled mimic peptide complex (M13-SA-HRP); the results of the peptide expression are presented in Figure S1. The SA-HRP complex catalyzes the oxidation of the TMB substrate, producing a measurable colorimetric signal [32]. In parallel, SERS signals were collected to enable quantitative bacterial analysis [33]. This dual-signal detection strategy not only significantly enhances sensitivity but also allows for rapid and reliable detection.

### 3.2. Synthesis and Characterization of the “Sandwich” Structure of IMBs–Bacteria–Phage

To visually examine the morphology of the MBs and IMBs, transmission electron microscopy (TEM) was employed. As characterized in our previous work [27], the pristine MBs exhibit a well-defined spherical morphology with uniform size and homogeneous distribution. After antibody conjugation (Figure S2), a distinct flocculent layer appears on the bead surface, indicating the successful immobilization of antibodies. Notably, the conjugation process does not induce any observable changes in the overall morphology of the magnetic beads.

Additionally, the micro-structure of the “sandwich” system developed in this study was analyzed by TEM (Figure 2). Figure 2a shows the IMBs-*Salmonella typhimurium* complex, where a significant number of IMBs are clearly attached to the surface of the *Salmonella typhimurium* cells. Figure 2b shows the TEM image of the IMBs@*Salmonella typhimurium*@M13 bacteriophage “sandwich” structure, where a distinct layer of M13 bacteriophage is observed on the surface of *Salmonella typhimurium* cells. This observation not only confirms the successful binding of M13 bacteriophage to *Salmonella typhimurium*, but also demonstrates the successful construction of the IMBs–bacteria–bacteriophage “sandwich” structure, which is crucial for the detection process.

As shown in Figure 2c, the reaction system containing the “sandwich” structure exhibits a pronounced absorption peak at 641 nm, corresponding to the formation of blue ox-TMB. In contrast, no evident absorption peak at 641 nm is observed in the other five control reaction systems. These results demonstrate that the presence of the “sandwich” structure markedly promotes the oxidation of TMB to generate blue ox-TMB. Consequently, the IMB + Sal + M13 + SA-HRP + TMB reaction system is validated as an effective platform for the colorimetric detection of *Salmonella typhimurium*. Importantly, even with steric hindrance, the successful signal amplification shown in Figure 2c empirically confirms that a sufficient number of SA-HRP molecules bind to the phage to generate a detectable enzymatic response, regardless of the exact occupancy number.

During Raman analysis (Figure 2d), the gold–magnetic composite prepared in previous experiments was employed as the SERS substrate. In the presence of the “sandwich” structure, a strong Raman signal characteristic of ox-TMB was clearly observed. In contrast, the other five control reaction systems exhibited no distinct Raman peaks, showing only weak background signals, which can be attributed to the limited Raman response associated with TMB oxidation. These results indicate that the IMB+Sal+M13+SA-HRP+TMB reaction system is suitable for the SERS-based detection of *Salmonella typhimurium*.

### 3.3. Optimization of the “Sandwich” Detection System of IMBs-Bacteria-Phages

To optimize the detection performance of the “sandwich” structure, key parameters of the IMB+Sal+M13+SA-HRP+TMB reaction system were systematically investigated. As shown in Figure 3b, the absorbance at 641 nm in the colorimetric assay increased progressively with reaction time, reaching a maximum at 16 min, followed by a gradual decrease. Accordingly, a reaction time of 15 min was selected as the optimal condition for colorimetric detection. For the Raman-based assay, the Raman intensity at 1602 cm^−1^ increased with reaction time; however, the rate of signal enhancement gradually diminished. Based on these observations, 20 min was determined to be the optimal reaction time for SERS detection.

Reaction temperature is a critical parameter influencing the catalytic activity of the enzyme in the “sandwich” structure. In the one-pot procedure, the concentration of *Salmonella typhimurium* was adjusted to 1 × 10^5^ CFU/mL under sterile conditions, followed by the sequential addition of predetermined amounts of IMBs, M13 bacteriophage, and SA-HRP. The mixture was then diluted to a final volume of 1 mL with sterile PBS (pH 7.4, 0.01 M). The effect of incubation temperature (25 °C, 30 °C, 35 °C, 37 °C, 40 °C, 43 °C, and 45 °C) on the enzymatic activity of the “sandwich” structure was systematically evaluated. As shown in Figure 3c, the highest colorimetric response, Raman signal intensity, and capture efficiency were obtained at 37 °C, indicating optimal catalytic performance. Accordingly, 37 °C was selected as the optimal reaction temperature.

In addition, the reaction time in the one-pot procedure is another critical parameter affecting the enzymatic catalytic activity of the “sandwich” structure. The influence of incubation time, ranging from 10 to 90 min, on the enzymatic catalytic performance of the “sandwich” structure was systematically investigated. As shown in Figure 3a, the colorimetric response, Raman signal intensity, and capture efficiency all reached their maximum values at an incubation time of 80 min, indicating optimal catalytic activity. Accordingly, 80 min was selected as the optimal reaction time.

Furthermore, the specificity of the IMB + Sal + M13 + SA-HRP + TMB reaction system was systematically evaluated. *Salmonella typhimurium* was tested at a concentration of 1 × 10^4^ CFU/mL, whereas other interfering bacterial strains were examined at a higher concentration of 1 × 10^5^ CFU/mL. As shown in Figure 3d, the reaction system generated a pronounced response exclusively toward *Salmonella typhimurium*, while no significant signals were observed in the presence of other bacterial strains. These results demonstrate that the IMB + Sal + M13 + SA-HRP + TMB system possesses excellent specificity for the detection of *Salmonella typhimurium*.

### 3.4. Sensitivity of the “Sandwich” Structure Detection System of IMBs–Bacteria–Phages

Given the specific recognition capability of the M13 bacteriophage toward *Salmonella typhimurium*, it demonstrates considerable potential for the selective detection of *Salmonella typhimurium* across a wide range of applications [34,35].

In the colorimetric assay, as shown in Figure 4, as the concentration of *Salmonella typhimurium* increased, the solution color of ox TMB gradually changed to blue (Figure 4a). The absorbance at 641 nm of the IMB + Sal + M13 + SA-HRP + TMB reaction system gradually increased with increasing concentrations of *Salmonella typhimurium* ranging from 4 × 10^1^ to 4 × 10^7^ CFU/mL (Figure 4b). A good linear relationship was established between the logarithm of *Salmonella typhimurium* concentration and the absorbance at 641 nm. The calibration curve was fitted as *y* = 0.06377*x* + 0.11459 (R^2^ = 0.98486), where *x* represents the logarithmic concentration of *Salmonella typhimurium* and *y* denotes the corresponding absorbance at 641 nm (Figure 4c). Based on this calibration curve, the limit of detection (LOD) was calculated to be 3.4 CFU/mL (3σ/slope).

As shown in Figure 4d, the SERS intensity at 1602 cm^−1^ of the IMB+Sal+M13+SA-HRP+TMB reaction system gradually decreased with increasing concentrations of *Salmonella typhimurium* ranging from 4 × 10^1^ to 4 × 10^7^ CFU/mL. A good linear relationship was established between the logarithmic concentration of *Salmonella typhimurium* and the corresponding SERS intensity at 1602 cm^−1^. The calibration curve was fitted as *y* = 218.11818*x* − 222.05661 (R^2^ = 0.98133), where *x* denotes the logarithmic concentration of *Salmonella typhimurium* and *y* represents the SERS intensity at 1602 cm^−1^ (Figure 4e). Based on this calibration curve, the limit of detection (LOD) was calculated to be 2.7 CFU/mL (3σ/slope).

The results indicate that the IMB+Sal+M13+SA-HRP+TMB reaction system developed in this study exhibits high sensitivity and a relatively broad linear detection range for both colorimetric and SERS-based detection of *Salmonella typhimurium*.

Next, the reproducibility of the SERS signals generated by the IMB + Sal + M13 + SA-HRP + TMB reaction system during Raman detection was evaluated, which is a critical requirement for SERS-based applications. The reaction mixture was collected and deposited onto a glass slide, and Raman spectra were randomly acquired from 30 different locations across the sample surface. As shown in Figure S3a, the three-dimensional waterfall plot demonstrates a high degree of signal uniformity, with consistent spectral profiles and negligible variations in the intensities of the characteristic peaks. Figure S3b presents the histogram of the integrated SERS intensity at 1602 cm^−1^. The relative standard deviation (RSD) of the SERS peak intensity was calculated to assess experimental reproducibility and was determined to be approximately 13.47%, indicating that the constructed “sandwich” structure provides good reproducibility for *Salmonella typhimurium* detection.

To verify the reliability and stability of the SERS method for detecting ultra-low concentrations of *Salmonella typhimurium* (3.0 CFU/mL), the constructed “sandwich” system was applied for SERS analysis (Figure 5), and selective MacConkey agar plating was performed for colony enumeration. Briefly, 30 mL of a *Salmonella typhimurium* suspension with a final concentration of 3.0 CFU/mL was prepared and divided into two equal portions (10 mL each). Each 10 mL portion was subsequently subdivided into ten aliquots (1 mL each), and three independent parallel experiments were conducted.

As shown in Figure 5, across three independent experiments, the *Salmonella typhimurium* concentration per milliliter of sample, as determined by the plate count method, ranged from 0 to 4.2 CFU/mL. In comparison, the bacterial concentration quantified using the SERS method ranged from 0 to 2.35 CFU/mL, showing good agreement with the plate count results. These results demonstrate that the SERS-based approach enables stable and reliable detection of *Salmonella typhimurium* at single-digit CFU levels.

Additionally, the storage stability of the constructed “sandwich” structure was evaluated. As shown in Figure 6a, the enzymatic activity of the “sandwich” structure retained approximately 80% of its initial colorimetric response and SERS intensity after 14 days of storage, indicating good storage stability. Furthermore, the analytical performance of the method developed in this workwas compared with those of recently reported detection strategies. As shown in Figure 6b, the proposed method exhibits superior sensitivity for the detection of *Salmonella typhimurium*.

### 3.5. Practicality and Feasibility Analysis

The reliability and practical applicability of the proposed method were further validated using real food samples. *Salmonella typhimurium* was spiked into shrimp, beef, and peanut samples at various concentrations ranging from 5 × 10^4^ to 5 × 10^6^ CFU/mL.

The colorimetric detection results are summarized in Table S1. The spiked recovery rates of *Salmonella typhimurium* in *Penaeus vannamei*, beef, and peanut samples ranged from 98.8% to 122.4%, 98.4% to 119.6%, and 97.4% to 110.4%, respectively. The corresponding coefficients of variation (CVs) were 12.42–19.97%, 6.13–14.79%, and 8.76–15.94%, respectively.

The SERS-based detection results are summarized in Table S2. The spiked recovery rates of *Salmonella typhimurium* in *Penaeus vannamei*, beef, and peanut samples ranged from 99.6% to 108.6%, 109.0% to 120.8%, and 99.6% to 115.2%, respectively. The corresponding coefficients of variation (CVs) were 7.65–18.99%, 5.27–12.80%, and 4.90–10.47%, respectively.

These results demonstrate that the proposed method offers high analytical accuracy and is well suited for the detection of *Salmonella typhimurium* in real food samples.

## 4. Conclusions

In this study, a bifunctional M13 phage was introduced to specifically recognize *Salmonella typhimurium* while simultaneously amplifying the enzyme loading. Combined with a magnetic separation-based immunoassay and dual-signal readout (colorimetric and SERS), the developed method enabled the detection of *Salmonella typhimurium* at the single-bacterium level. Furthermore, this site-directed engineering approach confers strong programmability and generalisability: by changing the genetically modified site or displayed recognition element, the same platform can be extended to other targets. The proposed method holds promising potential for diverse field applications, particularly in food safety monitoring (e.g., dairy products, eggs, meat, and ready-to-eat foods), clinical diagnostics (e.g., stool and blood samples for point-of-care testing), and environmental surveillance (e.g., drinking water and aquaculture systems). Although the total assay time is approximately 1–2 h, the hands-on time is considerably shorter, making the method suitable for routine screening in resource-limited settings. Nevertheless, several limitations still exist in the present study, such as the steric hindrance issue, which requires precise experimental determination of the number of SA-HRP molecules loaded per M13 phage to quantify the signal amplification factor, as well as the specificity against other *Salmonella serotypes*. These aspects will be further investigated in our future studies.

## Figures and Tables

**Figure 1:**
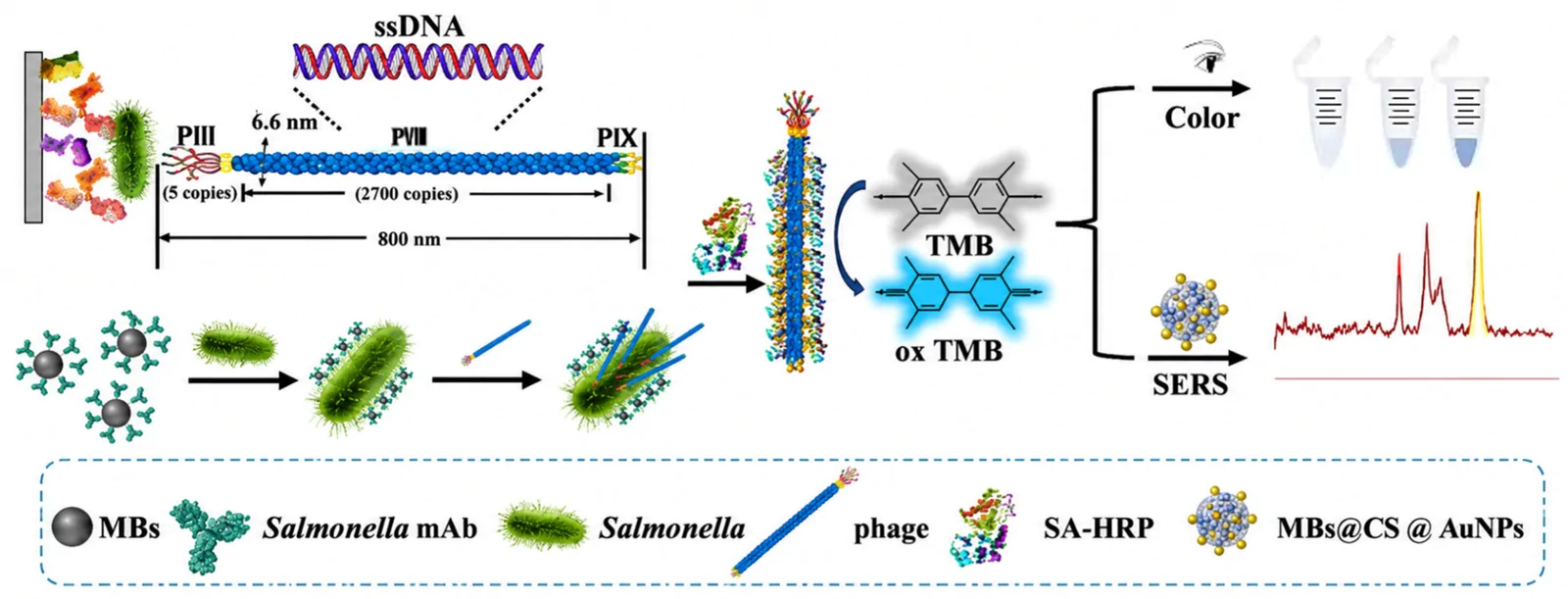
Schematic diagram of the magnetic bead–bacteria–phage “sandwich”-structured dual-signal method for quantitative detection of *Salmonella typhimurium*.

**Figure 2:**
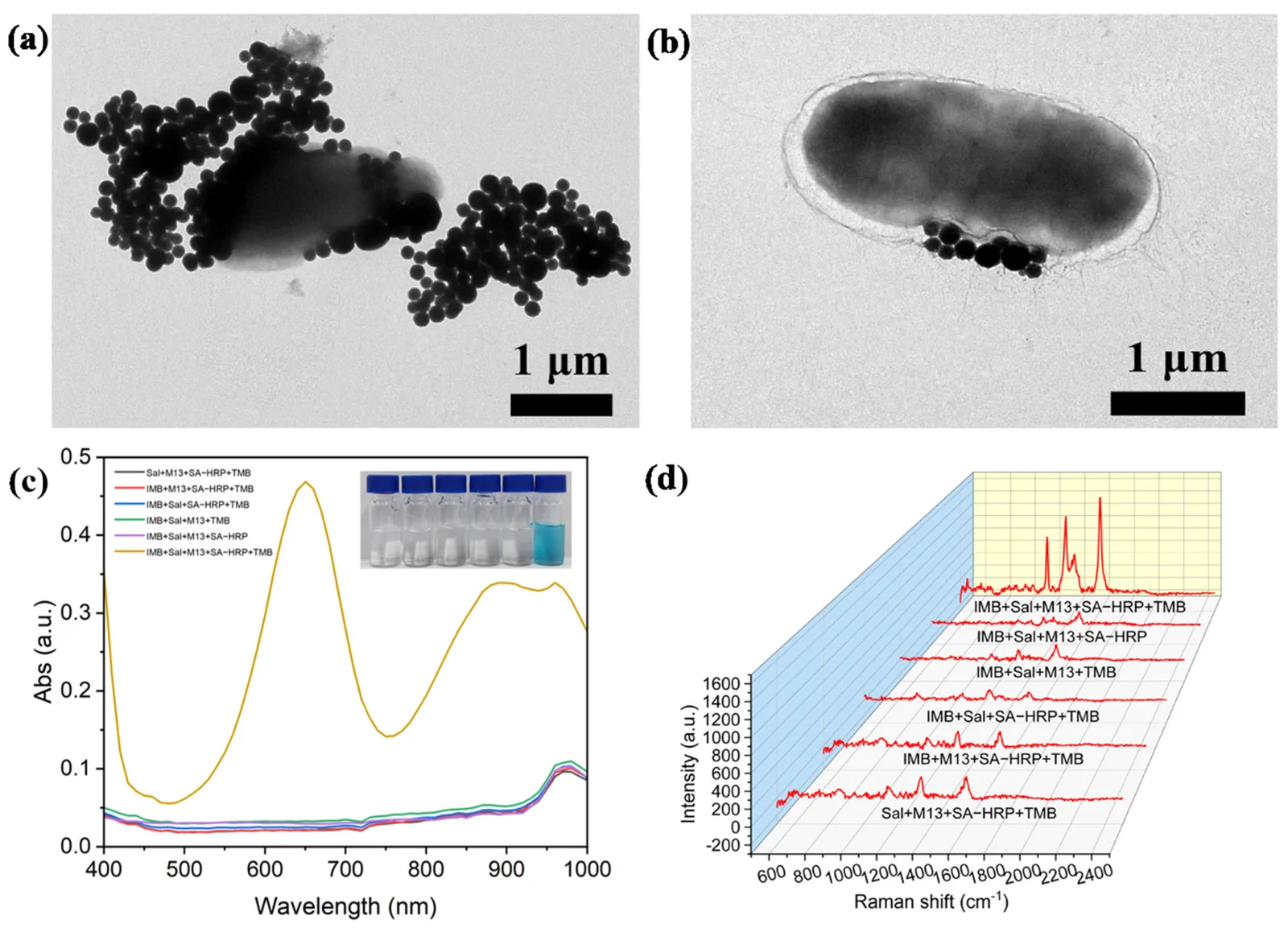
(**a**) TEM image of immunomagnetic beads (IMB) capturing *Salmonella*; (**b**) TEM image of the “sandwich” structure formed by IMB capturing *Salmonella* and coupling with phages; (**c**) UV-vis characterization diagram; (**d**) Raman characterization diagram.

**Figure 3:**
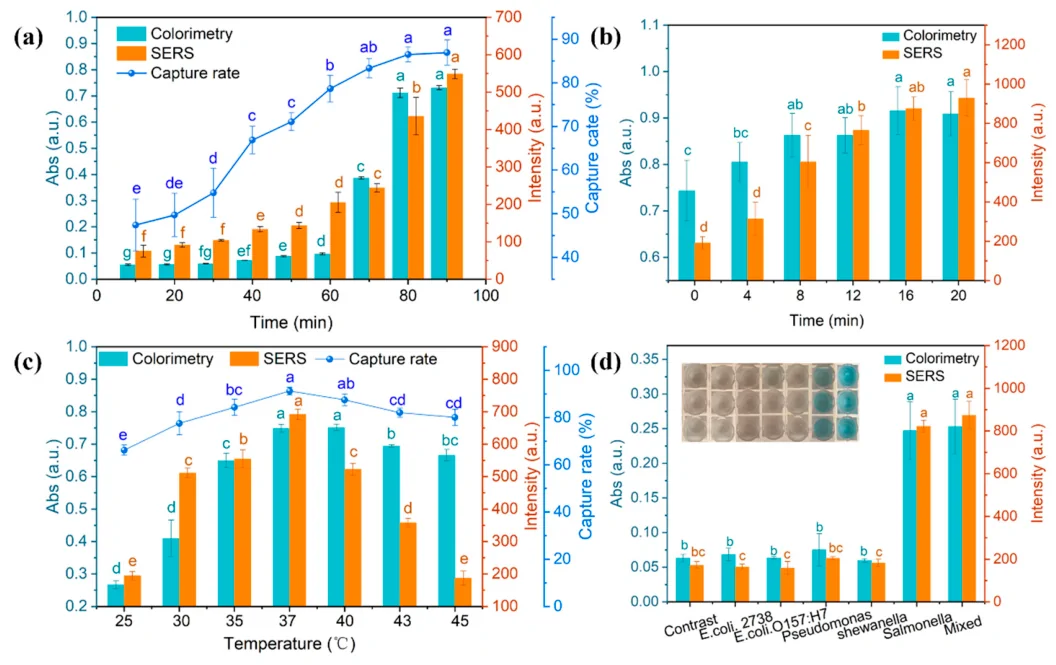
(**a**) Optimization of the reaction time in the “one-pot” method, (**b**) optimization of the TMB display time, (**c**) optimization of the temperature, and (**d**) specificity of the dual-signal system. Different lowercase letters (a, b, c, d...) above the bars indicate significant differences between groups (*p* < 0.05). Groups sharing the same letter or containing a common letter are not significantly different.

**Figure 4:**
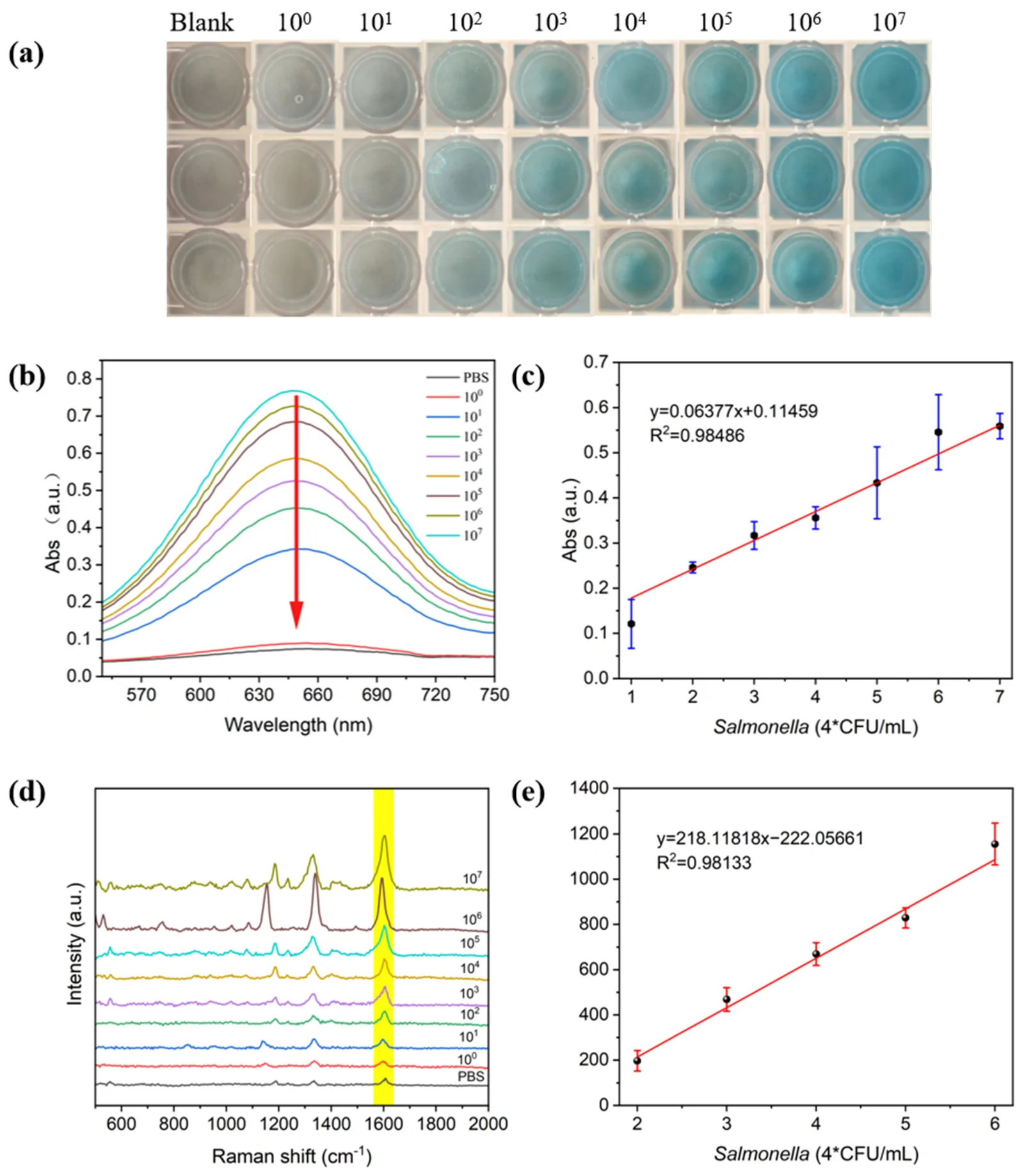
(**a**) Images of TMB chromogenic reaction with the *Salmonella* concentration ranging from 4 × 10^0^ to 4 × 10^7^ CFU/mL; (**b**) UV-vis absorption spectrum diagram; (**c**) calibration curves of *Salmonella* in the colorimetric mode; (**d**) SERS spectra; (**e**) calibration curves of *Salmonella* in the SERS mode.

**Figure 5:**
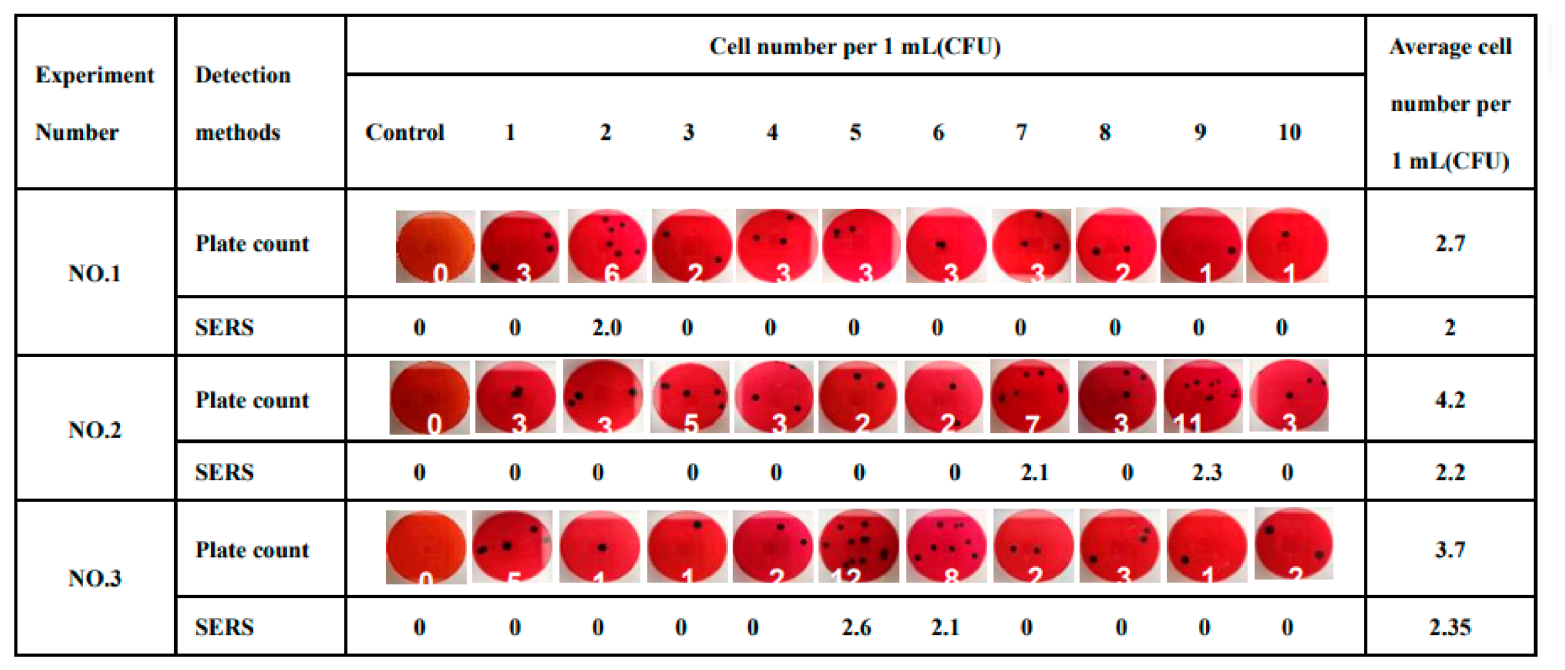
Evaluation of the reliability of the SERS mode in the detection of ultra-low concentrations of *Salmonella* (3 CFU/mL).

**Figure 6:**
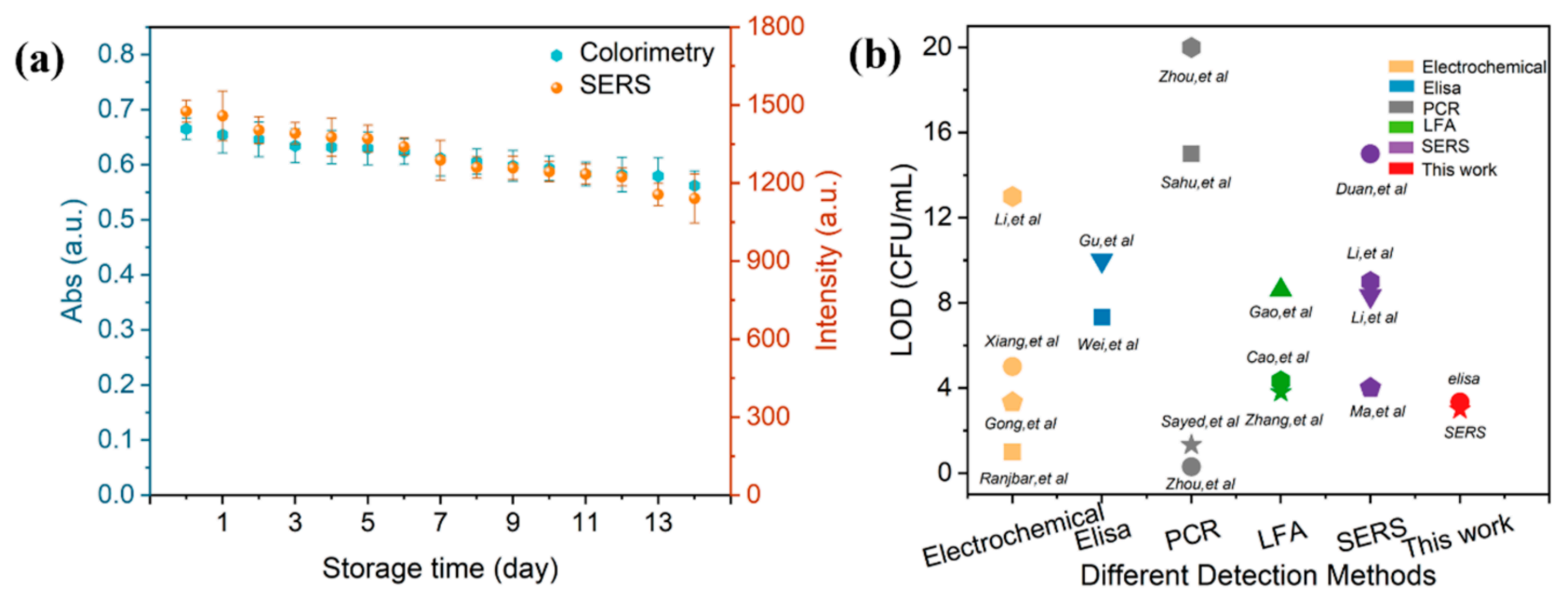
(**a**) Stability. (**b**) Comparison with other methods.

## Data Availability

The original contributions presented in the study are included in the article/Supplementary Materials. Further inquiries can be directed to the corresponding author.

## Supplementary Materials

The following supporting information can be downloaded at https://www.mdpi.com/article/10.3390/foods15203681/s1: Figure S1. The confirmation result of peptide expression on M13 phage. Figure S2. TEM characterization diagrams: Immunomagnetic beads. Figure S3. (a) 3D SERS spectral waterfall plot of 104 M Salmonella obtained from 30 distinct positions on the substrate. (b) Histogram showing the integrated intensity of the 1602 cm^−1^ peak. Table S1. Recovery performance of the Salmonella assay in Penaeus vannamei, beef and peanut samples under colorimetric mode. Table S2. Recovery performance of the Salmonella assay in Penaeus vannamei, beef and peanut samples under SERS mode.
