# Supplementary material for: M13 Bacteriophage-Based Sandwich Assay for Salmonella Detection: A Dual-Signal Approach Combining Colorimetric and SERS Techniques
Source: Foods. 2026 Oct 16;15(20):3681. doi: 10.3390/foods15203681 (PMC13703598; doi:10.3390/foods15203681)
Supplement: Supplementary file 1 [file foods-15-03681-s001.zip › foods-4533230-supplementary.pdf]

Supplementary Information

M13 bacteriophage-based sandwich assay for *Salmonella* detection: A dual-signal approach combining colorimetric and SERS techniques

Shengnan Zhan[a], Yanke Wang[a], Yihui Yang[a], Qi Chen[b], Xizhi Shi[c], Hui Wang[c], Wenhua Yang[d], Xin Zhang[a], Tao Huang[a*]

[a] Zhejiang–Malaysia Joint Research Laboratory for Agricultural Product Processing and Nutrition, College of Food Science and Engineering, Ningbo University, Ningbo, Zhejiang Province, 315211, China
[b] National Engineering Research Center for Bioengineering Drugs and the Technologies, Jiangxi Provincial Key Laboratory of Bioengineering Drugs, Institute of Translational Medicine, Jiangxi Medical College, Nanchang University, Nanchang, 330031, China
[c] Key Laboratory of Aquacultural Biotechnology (Ningbo University), Ministry of Education, School of Marine Sciences, Ningbo University, Ningbo 315211, China
[d] State Key Laboratory of Food Science and Resources, Nanchang University, Nanchang 330047, China
[e]Key laboratory of Processing and Nutritional Health of Tropic and Subtropical Aquatic Specialty Foods, Guang Zhou, 510380, China

* Correspondence: author: Tao Huang, huangtao@nbu.edu.cn

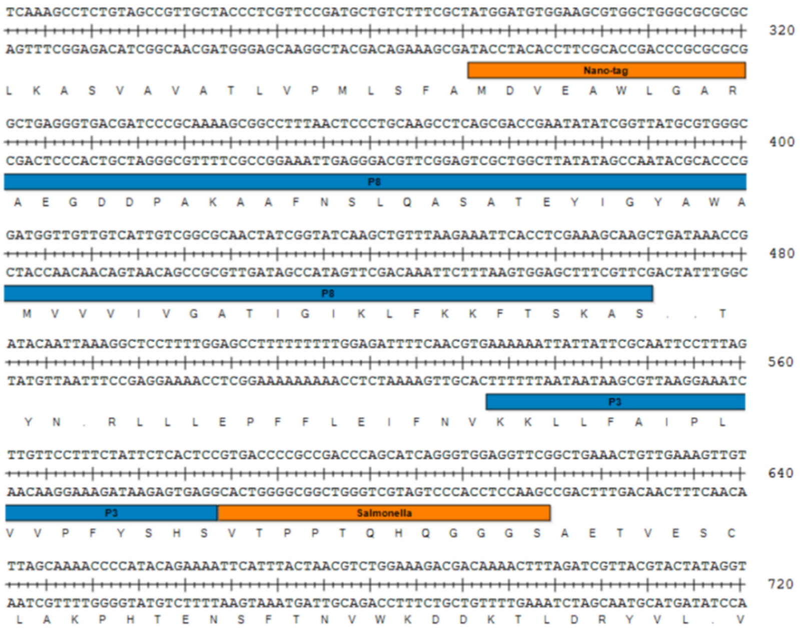

Figure S1. The confirmation result of peptide expression on M13 phage

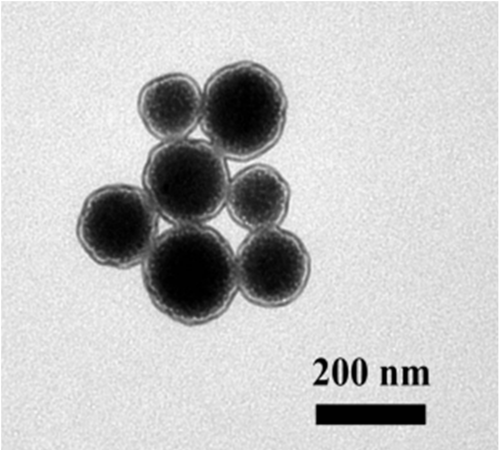

Figure S2. TEM characterization diagrams: Immunomagnetic beads

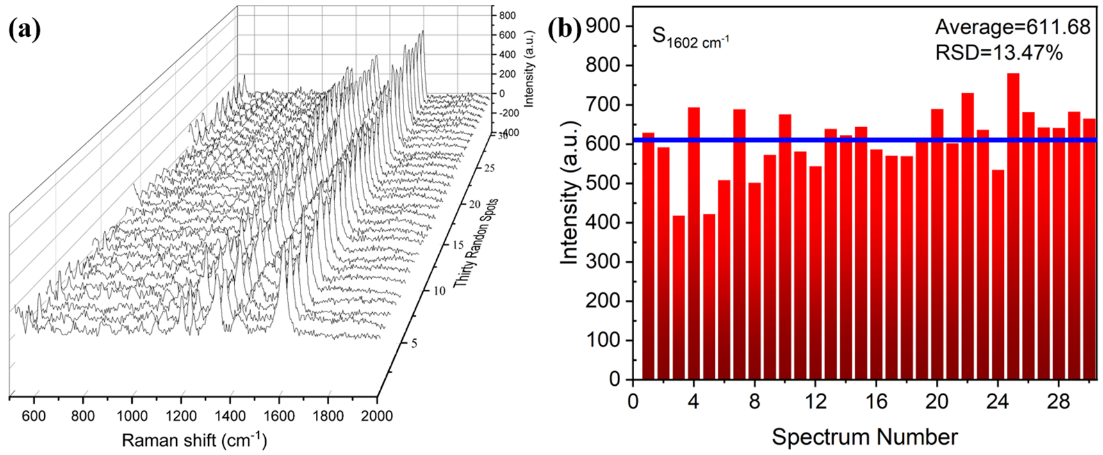

Figure S3. (a) 3D SERS spectral waterfall plot of 10^4 M *Salmonella* obtained from 30 distinct positions on the substrate. (b) Histogram showing the integrated intensity of the 1602 cm^{-1} peak.

Table S1. Recovery performance of the *Salmonella* assay in *Penaeus vannamei*, beef and peanut samples under colorimetric mode.

Salmonella added (CFU/mL)	Absorbance (a.u.)	Detecting concentration (CFU/mL)	Recovery (%)	CV (%)
***Penaeus vannamei* samples**				
$5×10^4$	0.482	$4.94×10^4$	98.8	19.972
$5×10^5$	0.554	$5.38×10^5$	107.6	15.387
$5×10^6$	0.627	$6.12×10^6$	122.4	12.423
**Beef samples**				
$5×10^4$	0.480	$5.98×10^4$	119.6	6.133
$5×10^5$	0.546	$5.78×10^5$	115.6	11.870
$5×10^6$	0.607	$4.92×10^6$	98.4	14.797
**Peanut sample**				
$5×10^4$	0.484	$4.87×10^4$	97.4	8.764
$5×10^5$	0.554	$5.16×10^5$	103.2	15.942
$5×10^6$	0.625	$5.52×10^6$	110.4	11.162

Table S2. Recovery performance of the *Salmonella* assay in *Penaeus vannamei*, beef and peanut samples under SERS mode.

Salmonella added (CFU/mL)	Raman intensity (a.u.)	Detecting concentration (CFU/mL)	Recovery (%)	CV (%)
***Penaeus vannamei* samples**				
$5×10^3$	258.973	$5.28×10^3$	105.6	18.99
$5×10^4$	391.134	$4.98×10^4$	99.6	11.47
$5×10^5$	531.873	$5.43×10^5$	108.6	7.65
**Beef samples**				
$5×10^3$	262.017	$5.45×10^3$	109	12.80
$5×10^4$	473.859	$5.72×10^4$	114.4	7.14
$5×10^5$	686.249	$6.04×10^5$	120.8	5.27
**Peanut sample**				
$5×10^4$	378.227	$4.83×10^3$	99.6	10.47
$5×10^5$	590.607	$5.44×10^4$	108.8	7.35
$5×10^6$	797.569	$5.76×10^5$	115.2	4.90
